# Supplementary material for: Multi-Dimensionality Immunophenotyping Analyses of MAIT Cells Expressing Th1/Th17 Cytokines and Cytotoxic Markers in Latent Tuberculosis Diabetes Comorbidity
Source: Pathogens. 2022 Jan 12;11(1):87. doi: 10.3390/pathogens11010087 (PMC8777702; doi:10.3390/pathogens11010087)
Supplement: Supplementary file 1 [file pathogens-11-00087-s001.zip › pathogens-1515156 - Supplementary Materials Figure.pdf]

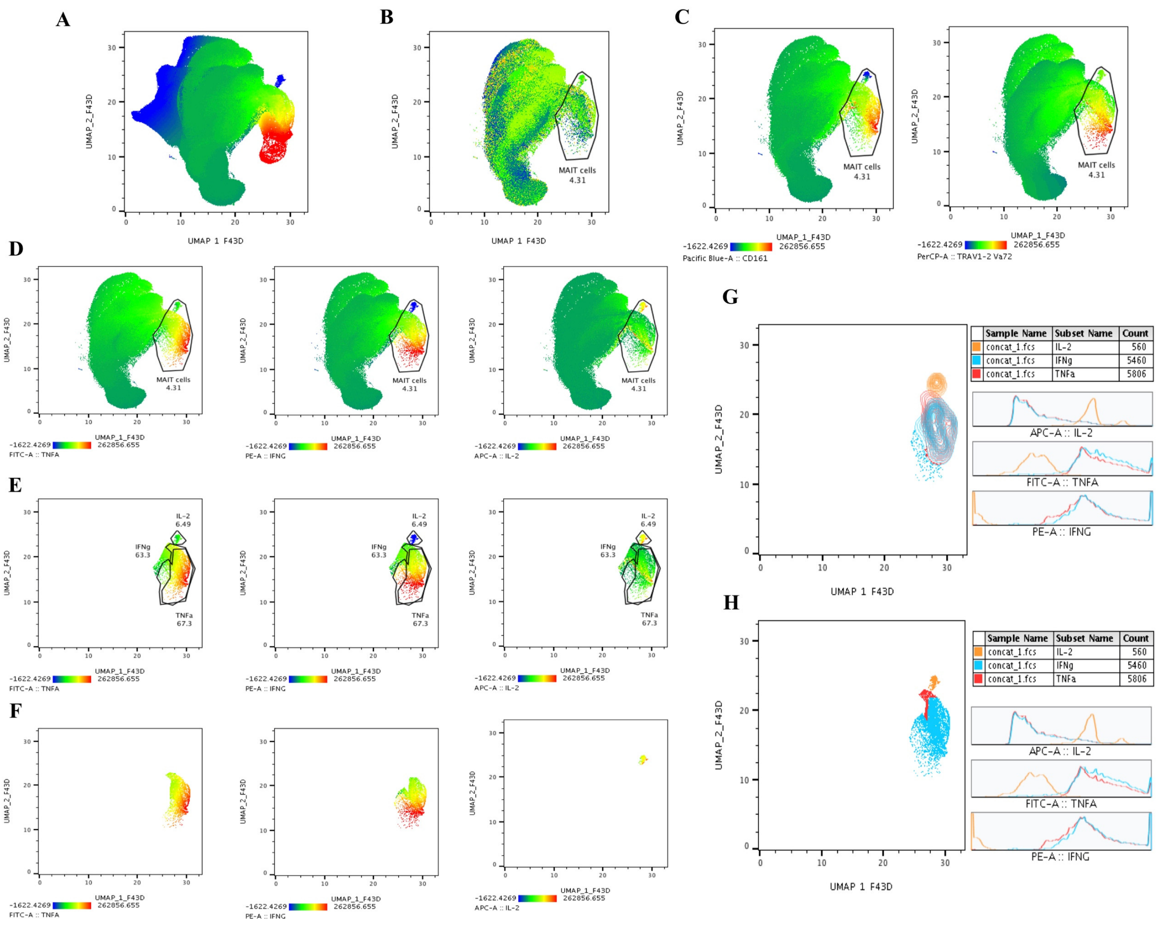

Figure S1: Multi-dimensionality profile of MAIT cells expressing Th1 (TNFα, IFNγ, and IL-2) cytokines using UMAP analysis.

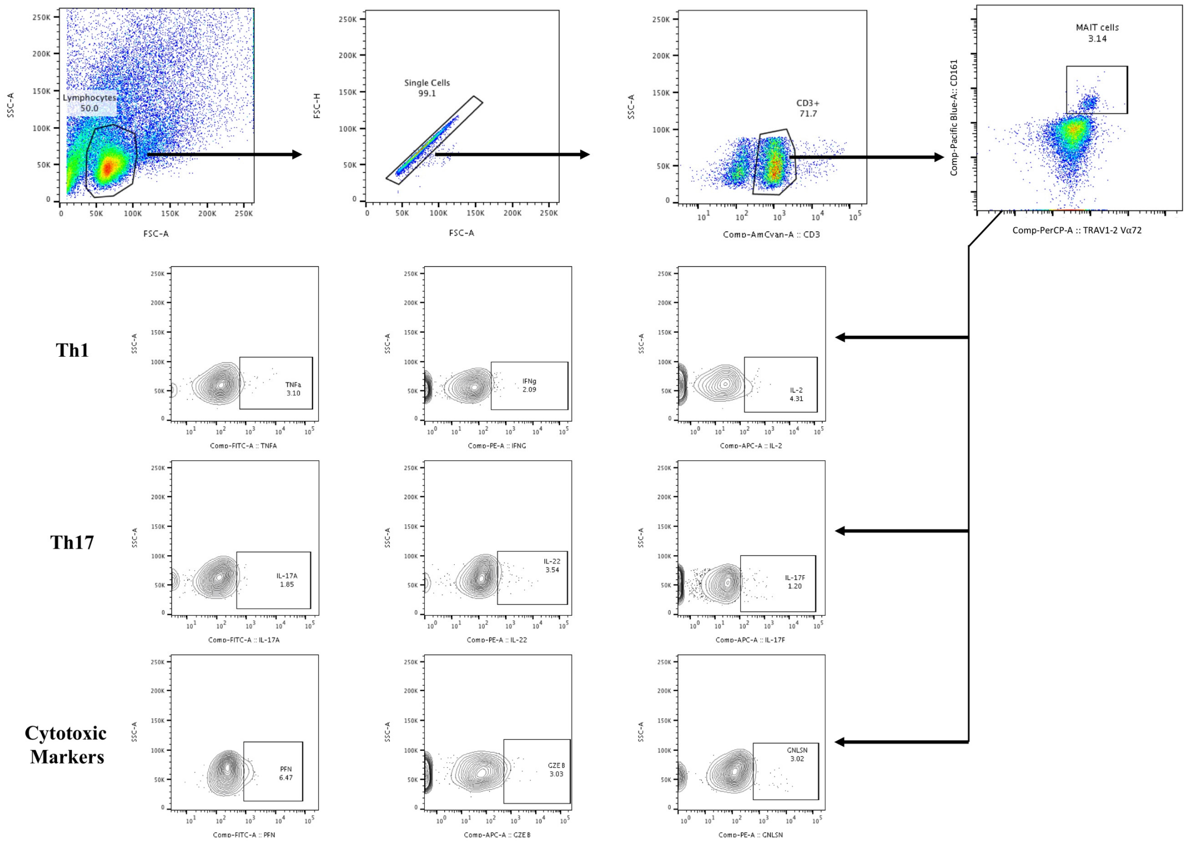

Figure S2: PBMCs' gating strategy and representative plots for MAIT cells expressing Th1 and Th17 cytokines, and cytotoxic markers

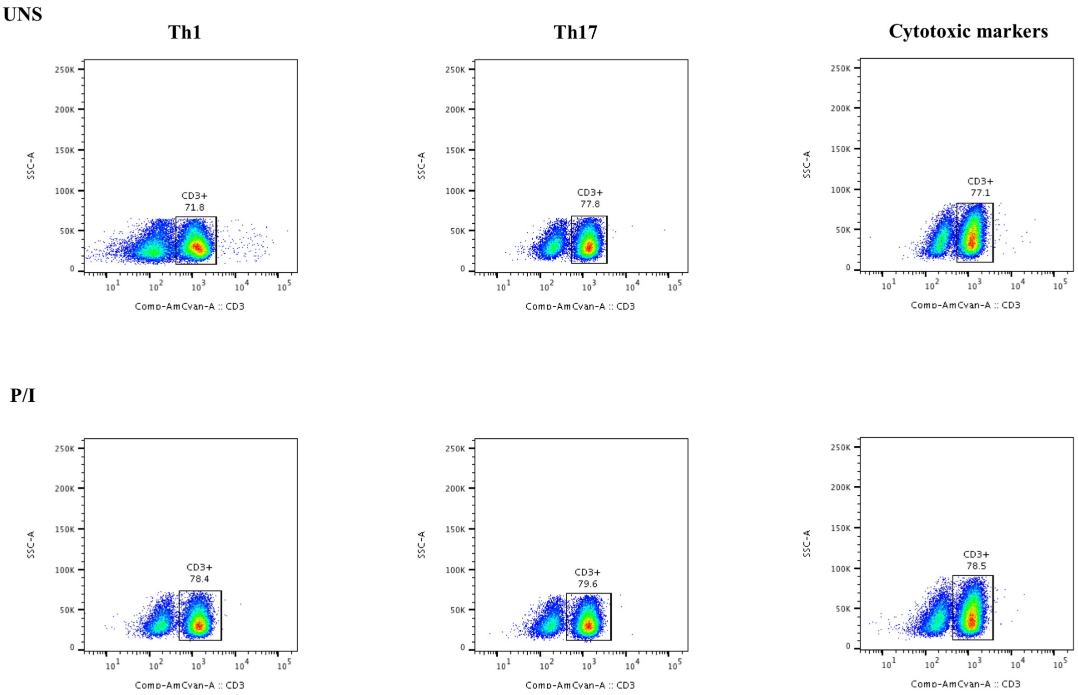

Figure S3: The percentage of $CD3^{+}$ population between unstimulated and P/I antigen stimulation.

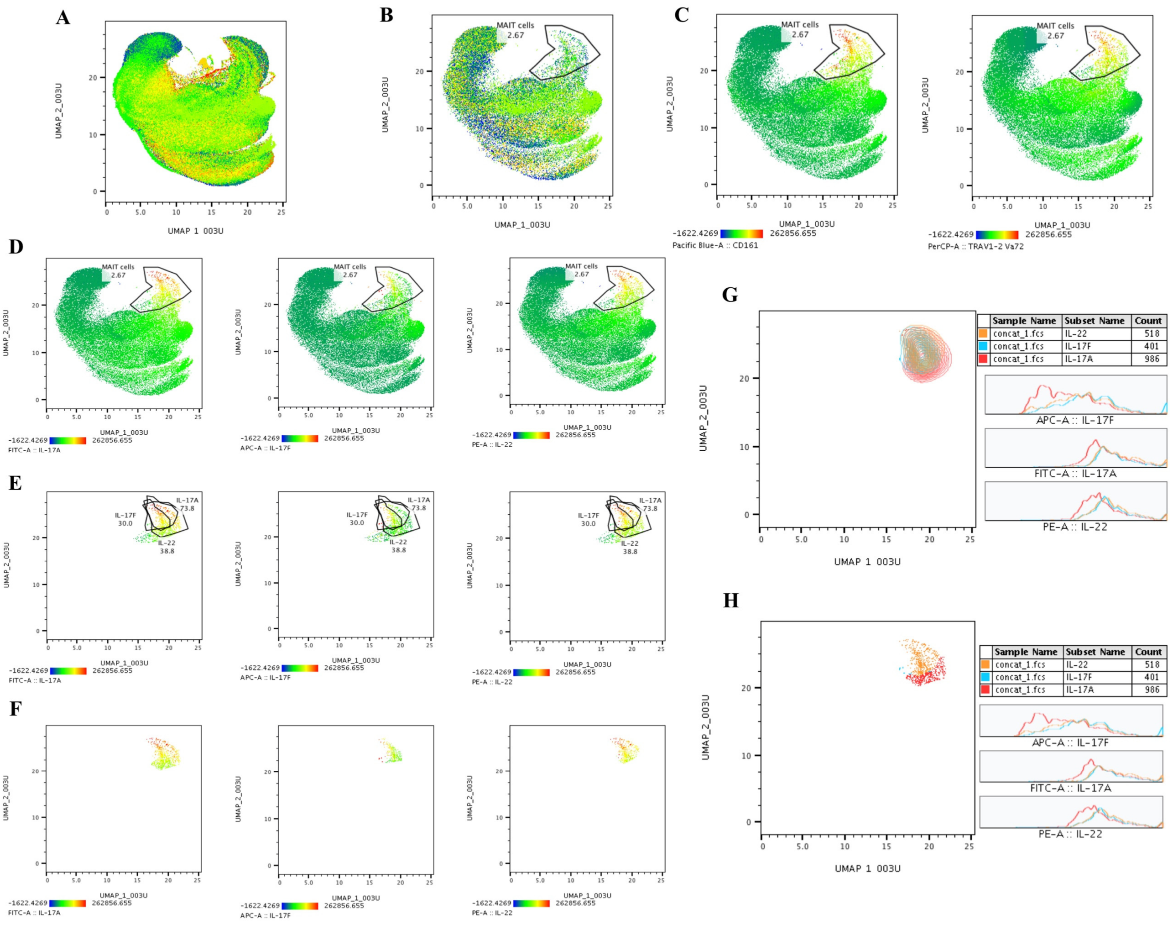

Figure S4: Multi-dimensionality profile of MAIT cells expressing Th17 (IL-17A, IL-17F, and IL-22) cytokines using UMAP analysis

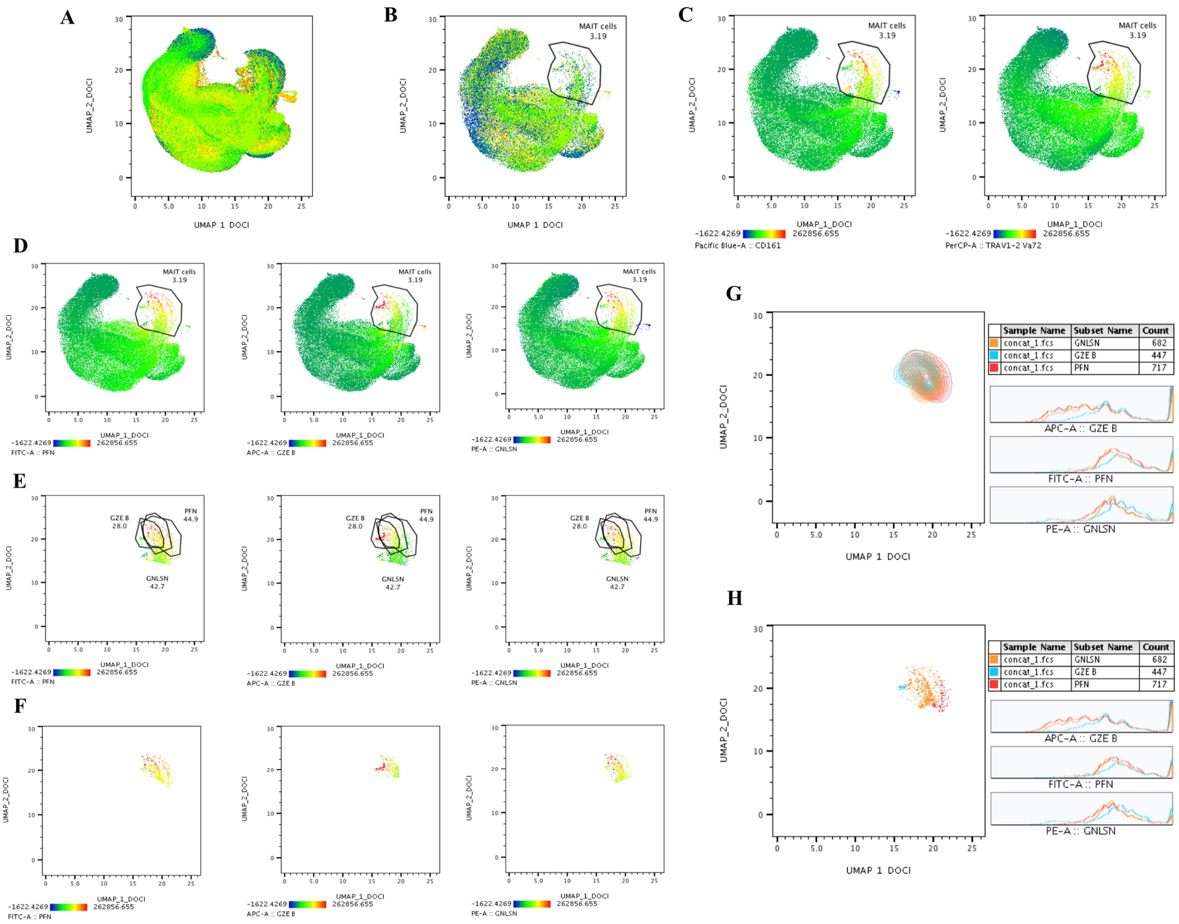

Figure S5. Multi-dimensionality profile of MAIT cells expressing cytotoxic markers using UMAP analysis. (A) Ungated UMAP profile of $CD3^{+}$ lymphocyte population (B) Overall MAIT cell gated population (TRAV1-2 Vα7.2 and CD161) (C) individual expression of TRAV1-2 Vα7.2 and CD161 markers (D) UMAP profile showing the expression of cytotoxic markers expressing MAIT cells (E, F) Overall and individual expression levels of cytotoxic markers frequencies (G, H) Histogram profile or dot blot expression of cytotoxic markers expressing MAIT cells.
